# Losing a jewel—Rapid declines in Myanmar’s intact forests from 2002-2014

**DOI:** 10.1371/journal.pone.0176364

**Published:** 2017-05-17

**Authors:** Tejas Bhagwat, Andrea Hess, Ned Horning, Thiri Khaing, Zaw Min Thein, Kyaw Moe Aung, Kyaw Htet Aung, Paing Phyo, Ye Lin Tun, Aung Htat Oo, Anthony Neil, Win Myo Thu, Melissa Songer, Katherine LaJeunesse Connette, Asja Bernd, Qiongyu Huang, Grant Connette, Peter Leimgruber

**Affiliations:** 1 Smithsonian Conservation Biology Institute, Conservation Ecology Center, Front Royal, Virginia, United States of America; 2 Department of Geosciences, University of Bayreuth, Bayreuth, Germany; 3 American Museum of Natural History, New York, New York, United States of America; 4 EcoDev/ALARM, Kamayut Township, Yangon, Myanmar; 5 GMAP, Hlaing Township, Yangon, Myanmar; University of Maryland at College Park, UNITED STATES

## Abstract

New and rapid political and economic changes in Myanmar are increasing the pressures on the country’s forests. Yet, little is known about the past and current condition of these forests and how fast they are declining. We mapped forest cover in Myanmar through a consortium of international organizations and environmental non-governmental groups, using freely-available public domain data and open source software tools. We used Landsat satellite imagery to assess the condition and spatial distribution of Myanmar’s intact and degraded forests with special focus on changes in intact forest between 2002 and 2014. We found that forests cover 42,365,729 ha or 63% of Myanmar, making it one of the most forested countries in the region. However, severe logging, expanding plantations, and degradation pose increasing threats. Only 38% of the country’s forests can be considered intact with canopy cover >80%. Between 2002 and 2014, intact forests declined at a rate of 0.94% annually, totaling more than 2 million ha forest loss. Losses can be extremely high locally and we identified 9 townships as forest conversion hotspots. We also delineated 13 large (>100,000 ha) and contiguous intact forest landscapes, which are dispersed across Myanmar. The Northern Forest Complex supports four of these landscapes, totaling over 6.1 million ha of intact forest, followed by the Southern Forest Complex with three landscapes, comprising 1.5 million ha. These remaining contiguous forest landscape should have high priority for protection. Our project demonstrates how open source data and software can be used to develop and share critical information on forests when such data are not readily available elsewhere. We provide all data, code, and outputs freely via the internet at (for scripts: https://bitbucket.org/rsbiodiv/; for the data: http://geonode.themimu.info/layers/geonode%3Amyan_lvl2_smoothed_dec2015_resamp)

## Introduction

Historically, Myanmar’s forests represented a resource coveted by local and foreign interests, ultimately leading to the invasion and colonization by the British [1,2]. To exploit Myanmar’s valuable teak, the British created the Burma Forest Department in 1856 and initiated scientific forest management which ensured and increased commercial hardwood production with little regard to the needs of Myanmar’s rural populations [1]. The ensuing conflict between the government and local people over the use of forest land and resources continues throughout Myanmar’s recent history [1]. However, Myanmar’s successful forestry sector provided an important basis for the country’s economic development, and after independence Myanmar became the second largest exporter of teak [3]. At the same time much of the country’s rural population continued to rely on forest resources to supplement their livelihoods.

As of 2010 nearly 70% of Myanmar’s population lived in rural areas [4]. Forest lands are used for small scale agroforestry, and up to 77% of the Myanmar’s energy demands are being met by traditional fuel sources, mostly fuelwood [5,6]. Although there have been frequent reports of mismanagement, overuse, and deforestation over the last three decades [7–10], past research indicated the country to be one of the most forested in the region [10]. For example, a countrywide analysis of Landsat data for the period 1990–2000 showed that Myanmar had retained much of its original forest cover, stretching across 65% of the country’s land [10].

Most of Myanmar’s remaining large forests are located in the far north and south of the country, connecting to other extensive forests in India and Thailand. The Northern Forest Complex stretches across Northern Sagaing and Kachin [11], linking up to forests in Assam and Arunachal Pradesh in India [10,11]. The Tanintharyi forests extend to Thailand’s Western Forest Complex and Kaeng Krachan National Park [12]. These areas are considered biodiversity hotspots [13] and are supporting many endangered species [14], including tigers (*Panthera tigris*), Asian elephants (*Elephas maximus*), Gurney’s pitta *(Pitta gurneyi)* and Asian tapir *(Tapirus indicus*) [15].

Myanmar retained these forests because of its long political and economic isolation from much of the world. However, this isolation is ending. The country’s recent political and economic reforms are attracting investors, leading to far-reaching changes in many sectors, including forestry and other land-based investments such as agro-businesses. These new developments place greater pressures on remaining forests [16]. Even areas previously inaccessible, due to armed conflicts between the government and ethnic groups, are starting to open up for systematic, large-scale resource extraction, timber production, and commercial plantations [15]. Non-agricultural sectors such as construction, mining and energy are increasingly contributing towards country’s GDP [17], and are impacting forests.

To assess how rapid economic and development changes are affecting Myanmar, new, accurate, and detailed information about the condition of remaining forests is urgently needed. Ideally, such assessments distinguish between intact and degraded forest, providing critical baseline data for biodiversity conservation. We ask following questions:

1. How much intact forest is remaining in Myanmar?
2. How fast is intact forest being converted?
3. Where are the forest loss hotspots in the country?
4. Where are the largest patches of remaining intact forest?

Myanmar’s intact forests have not been mapped previously. Our project is intended to increase access to this information and to expand technical capacity for forest mapping by civil organizations. We collaborated with national and international environmental NGOs, as well as research institution, to jointly develop methods that can be replicated and adapted for future forest mapping projects. We relied entirely on public domain data and open source software to facilitate maximum participation from local GIS teams and improve cost effectiveness. All data are being released with an open access license at http://geonode.themimu.info/layers/geonode%3Amyan_lvl2_smoothed_dec2015_resamp.

## Methods

### Study area

Myanmar (9°-29°N, 92°-102°E) is a Southeast Asian country that shares borders with Bangladesh, China, India, Laos, and Thailand. It has a high diversity of ecosystems, ranging from alpine grasslands in the north to tropical and mangrove forests in the south [15]. Large mountain ridges, covered with forests extend from the Himalayas, dividing the country into three major watersheds. Rain shadows from these mountains create a central dry zone where most of the population lives [15,18]. We conducted a forest cover change assessment for the whole country, with special focus on the most forested regions of Myanmar namely Tanintharyi, Kachin, and Sagaing.

### Mapping approach

Detecting spectral changes in satellite images collected on different dates is an effective strategy to map and quantify forest cover and change [19–22]. Forest loss leads to substantial changes in tree canopy cover which can be detected using high and medium (5-30m) resolution satellite data [23]. To identify and quantify changes in forest cover, we compared Landsat 5, 7, and 8 imagery for 2002 and 2014 (www.earthexplorer.usgs.gov). We collected the best available 104 Landsat scenes (cloud cover <10%, acquisition between December-February) covering 52 tiles (Paths: 129–135 and Rows: 40–53; S1 Table), using near anniversary date imagery, i.e. images collected during the same season and with similar phenology. Images acquired during the post-monsoon months from December to February are most useful for forest mapping in Myanmar, as canopies are lush during this time while cloud cover remains low [10].

To maximize our ability to differentiate land cover types, we used all Landsat bands that record surface reflectance in the visible, near- and mid-infrared spectrum and that have a 30-m resolution. This includes bands 1–5 and 7 for Landsat 5 and 7, and bands 1–7 and 9 for Landsat 8 imagery. These bands have been commonly used with Random Forest classifications in the past [24,25]. Rather than using specific band combination in maximum-likelihood classification, Random Forest models facilitate the use of the information contained in all band. More specifically, Random Forest models perform better than maximum-likelihood classifications by allowing for non-parametric and multi-model distribution across a multi-dimensional feature spaces. By including as many bands as possible, i.e. the entire multidimensional feature space of the satellite imagery, Random Forest provides for the detection and differentiation of even subtle spectral differences between land cover/land use categories that cannot be separate with maximum-likelihood classification of a few selected spectral bands.

For our classification, we analyzed Landsat images by tile location, allowing us to better account for differences in acquisition date and atmospheric conditions. This tile-by-tile approach enabled us to address gradual changes in forest types (e.g. lowland evergreen forest, mangroves, dry diperterocarp forest) across large environmental gradients (e.g. Himalayan foothills to Sundaic lowlands). These methods have been successfully applied in other studies and are widely used for land cover and land use mapping [22,26,27,28,29]. We included all spectral band information in the Random Forest model as predictive variables, and the variables were not weighted. The Random Forest model is capable of providing variable importance information, as well as feature space plots showing which values for each band characteristically corresponded to different land cover/land use types (see S1 Fig for example of a feature space plot). However, variable importance rankings and feature space plots vary among tiles and, consequently, we we do not provide individual or averaged importance rankings or feature space plots as these would not be very meaningful.

To aid with the identification of change in our bi-temporal Landsat data we applied the iteratively reweighted multivariate alteration detection (IR-MAD) algorithm (30) to all the bi-temporal Landsat data. IR-MAD is similar to other classification indices derived from Tassle-Cap or Principle Components Analysis (PCA) but is based on canonical correlation analysis (CCA) [30]. It identifies areas of change between the two image acquisition dates. This approach reduces the risk of confusing spectral changes resulting from different atmospheric conditions or sensor calibration with changes resulting from land cover/land use change [31]. We used this method to create a probability of no-change layer that was incorporated into the multi-date image stack and entered as another variable/band into our Random Forest classification.

We performed all analyses, image preprocessing, processing, and change detection in QGIS [32] and R statistical software [33], using remote sensing scripts (S1 File; https://bitbucket.org/rsbiodiv/). For better visual interpretation of changes in images and to verify training polygons during image classification, we relied on high spatial resolution imagery available through Google Earth and Bing Maps.

### Preprocessing

We calibrated all Landsat data to top of the atmosphere reflectance (TOA) values using radiometric rescaling coefficients provided in the Landsat metadata file (Landsat Level 1 Data Products, http://earthexplorer.usgs.gov/) [34,35]. Landsat 8’s OLI sensor includes an additional band (Band 1) which we excluded from our analysis to reduce noise resulting from high atmospheric scattering inherent with short wavelength image bands (Landsat Missions, http://landsat.usgs.gov/best_spectral_bands_to_use.php). To remove cloud and cloud shadow pixels and to improve forest cover classification, we used the stand-alone Function of Mask (Fmask) tool [36,37]. Fmask uses Landsat’s TOA reflectance and Brightness Temperature as inputs [36] and is designed to identify potential cloud pixels based on physical properties of clouds and parameters such as whiteness/darkness, temperature, thickness, reflectance, and Normalized Difference Vegetation Index (NDVI) [36].

### Forest classification

To decide on forest categories, definitions, and canopy cover thresholds for these categories, we conducted a 2-day, pre-mapping workshop with forest experts from government, universities, and conservation NGOs in Yangon in 2015. Based on the outcomes from this workshop we focused on 1) accurately delineating forests from other land cover and land use, 2) assessing forest condition based on canopy cover, and 3) quantifying conversion of forests into other land cover and land use categories when possible. We separated forest from water and all non-forest areas with less than 10% canopy cover (Table 1). The latter included all areas bare of vegetation (e.g. rock, bare soil, developed surfaces) and agriculture. Open-pit mining areas, which are concentrated in Kachin State and Saigang Region, could be identified because of their characteristic shape and proximity to rivers and were delineated separately from other non-forest areas. We then separated forests into intact forests, degraded forests, and plantations. Plantations, such as teak, oil palm, rubber, and sugar cane, can regularly be separated from other forests because of canopy structure, age uniformity, reduced undergrowth, and planting patterns that can be recognized in Google Earth imagery [38]. Additionally, we were able to differentiated oil palm from other types of plantations because of its characteristic canopy structure. Oil palm plantations were restricted to Tanintharyi because it is the only Myanmar region where climatic conditions support oil palm plantations [39,40].

**Table 1:** Land cover and land use categories used for forest mapping in Myanmar.

Category	Description
***Forest Cover***	
Intact Forest	>80% CC* in evergreen & mixed deciduous forests; >60% CC in dry deciduous forests**
Degraded Forest	10–80% CC in evergreen & mixed deciduous forests; 10–60% CC in dry deciduous forests
Water	Rivers, flooded river beds, rice paddies, lakes & reservoirs
Non-Forest	<10% CC
Plantations	Forest plantation (e.g. rubber, cashew)
Oil Palm Plantations	Oil palm plantation (Tanintharyi Region)
Mining	Mining (Kachin State and Sagaing Region)
Snow/Ice/Clouds	Snow, ice, as well as cloud holes which could not be fixed
***Forest Conversions***	
New Non-Forest	Intact forest in 2002 to <10% CC in 2014
New Mining	Intact forest in 2002 to mining in 2014 (Kachin State and Sagaing Region only)
New Plantations	Intact forest in 2002 to plantation in 2014
New Oil Palm Plantations	Intact forest in 2002 to oil palm plantation in 2014 (Tanintharyi Region only)
New Degraded Forest	Intact forest in 2002 to degraded forest in 2014
New Water	Reservoirs, changes in rivers, and hydro-electric projects

*CC = Canopy cover;

**restricted to forest found within Landsat tiles at Path: 132–134 and Row: 044–045.

We used an 80% canopy cover cut-off to separate between intact and degraded forests. To quantify forest conversion, we delineated all areas that transitioned from intact forest in 2002 to any other category. It is important to note that there are many land use and conversion activities that result in degraded forests and that we currently cannot separate from each other using Landsat satellite imagery in change detection. Degraded forests in our study represent areas where forest canopies have been reduced by relatively fine-scale and low-impact land use and conversion processes, including fine-scale logging and high-grading, fuelwood harvest, fine-scale permanent agriculture (often including cash crops such as rubber, bananas, sugar cane), and shifting agriculture. Although our study can provide an overall estimate of the combined effect of these activities on forest declines, it is impossible to measure the relative importance.

Our tile-based classification approach allowed us to adjust canopy cover cut-offs for dry dipterocarp forests, a rare and endangered forest type in Southeast Asia, characterized by open canopies. These forests are limited to areas with distinct dry season, limited rainfall, and low elevation. What little remains in Myanmar is found at the edges of the horseshoe-shaped dry zone the land transitions into hill country, the foothills of the Himalayas extending in two long mountain chains from the north of Myanmar to the south [41,42,43,44]. To ensure inclusion of these forests in our intact forest category, we relaxed our canopy cover cut-off to 60% for 7 Landsat tiles (Path-Row: 131045, 132044, 132045, 133044, 133045, 134044, 134045) covering southern Sagaing region and western-central Shan state where dry dipterocarp forests are common [41,42,43,44]. Although Landsat footprints do not conform to the exact climatic or elevational extent of dry dipterocarp forests, these Landsat tiles contain most of Myanmar’s remaining dry dipertocarp forest.

Google Earth, and other virtual globe mapping systems, have been widely used for data collection and validation in remote sensing projects [45,46,47], because they facilitate collection of independent data for training and for accuracy assessments in remote sensing in cases where field data collection is logistically difficult, expensive, and potentially dangerous. We used Google Earth and other high resolution webGIS layers to generate training data for forest classification. In addition, we utilized information based on a global forest cover loss product produced by Hansen et al 2013 [26]. The latter is a useful cross-check for determining whether canopy cover changes determined by Landsat could also be detected in high-resolution imagery in Google Earth and vice versa. Analysts manually digitized between 10–30 training polygons (Minimum Mapping Unit = 5 ha) delineating homogeneous areas for each of our land cover/land use categories. Training polygons were distributed across the entire satellite image to capture the full spectral variation for categories across the entire Landsat tile. Analyst also took special care to include training polygons representing different slopes and aspects to reduce problems from topographic shading. We then extracted spectral characteristics for each mapping category from satellite imagery using these training polygons

We performed supervised classification using the Random Forest in R [48,49], a non-parametric machine-learning algorithm that is widely applied for remote sensing [50], species distribution modeling, and other classifications [51]. Random Forest can utilize a large number of predictive variables (e.g. spectral bands) and it is especially suited for land cover classification because the model is robust even when the predictive variables share significant covariance and follow non-normal distributions. One of the main advantages of the Random Forest model is that it is an ensemble tree model, i.e. the results of large number of trees are aggregated internally. For classification models, the output is the class that receives the majority vote. These widely-used ensemble models have a much higher accuracy than any single tree classifier. We selected a large number of trees (ntree) to ensure that the Random Forest results converge. This means that, given the same dataset, different Random Forest runs will provide virtually identical results and the results of our final classification are therefore reproducible.

We classified different land cover types using reflectance information from all spectral bands included. We mosaicked all classified scenes into a single forest cover/forest conversion map.

Cloud cover in Landsat scenes can make remote sensing challenging [23]. To fill large cloud and cloud shadow gaps (≥100 ha) we downloaded and classified additional Landsat imagery for time periods close to original image date. For small cloud gaps (<100 ha), we manually digitized forest cover classes using Google Earth and Bing Maps.

### Accuracy assessment

The objective of our accuracy assessment was to evaluate ability for detecting and delineating intact forest. Rather than assessing pixel-based accuracies, we wanted to know how often we missed important patches of intact forests. For our ground-truth we relied on available high resolution imagery from Google Earth. This meant we had to limit our accuracy assessment to the 2013–2014 period because we could not find sufficient high resolution imagery for the 2002–2003, and consequently could also not assess accuracy in mapping changes. We vectorized our raster map, and used stratified random sampling to select 2,200 polygons based on major category (intact forest, degraded forest, non-forest, plantation) and patch size (1–100 ha, 300 polygons per class; 100–500 ha, 200 polygons per class; 500–500 ha, 50 polygons per class). We stripped the polygons of their class assignment to allow for an unbiased and blind assessment by our analysts. Analyst then overlaid the polygons in Google Earth and assessed the availability of high resolution imagery for 2013–2014, working simultaneously through the three size categories with polygons assessed in random order. When the team reached ~1,000 polygons inspected, we decided there was a reasonable sample size for the accuracy assessment. Polygons that did not overlay with high resolution imagery were discarded. The remaining 540 polygons were then visually assigned to a majority forest cover category observed within the polygon (intact forest, degraded forest, non-forest, and plantation). We compared assignments to classifications in an error matrix.

## Results

### Countrywide forest cover change

**Fig 1:**
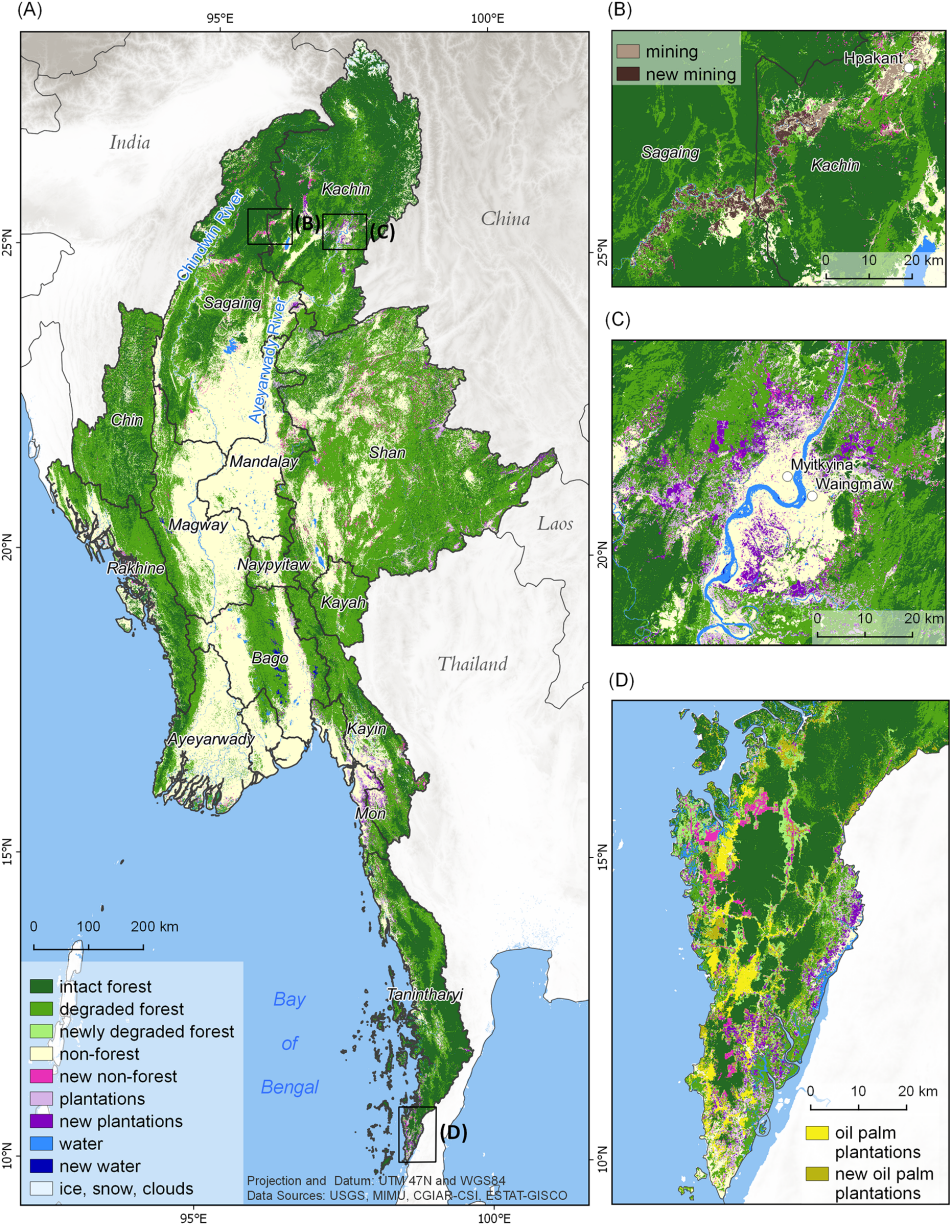
Distribution of forest cover and forest cover change across Myanmar. (A) Countrywide forest cover and change; (B) Forest losses from mining along the Uru River in Kachin and Sagaing; (C) Plantation development near Myitkyina, Sagaing; (D) Plantation development near Mawdaung, Tanintharyi.

In 2014, Myanmar’s forests covered 42,365,729 ha or 63% of the country’s terrestrial surface (Table 2, Fig 1), making Myanmar one of the most forested countries in the region. Our change analysis shows that forest cover declined by more than 1.5 million ha from nearly 44 to 42 million ha, with an annual net loss of 0.30% between 2002 and 2014. Intact forest cover is declining more rapidly than overall forest cover, and makes up only about 16 million ha, equivalent to 24% of the land area and 38% of Myanmar’s forest cover (Table 2). More than 2 million ha of forest loss was from intact forest, reducing these valuable ecosystems by >11%, with an annual net loss rate of 0.94%. Forest losses can be explained through conversion to (Table 2; Fig 1):

- Degraded forests resulting from overuse for logging, fuelwood consumption, and shifting cultivation (0.47 million ha)
- Other non-forest land uses such as mining, clear-cutting for agriculture, and infrastructure (1.00 million ha).
- Plantation crops such as oil palm, rubber, and sugar cane (0.54 million ha)
- Hydro-electric dams and reservoirs (0.07 million ha).

**Table 2:** Country-wide forest cover change in Myanmar between 2002 and 2014.

Category*	Cover [ha]		Change		Annual Net Change
	2002	2014	[ha]	%	%
Forest**	43,962,152	42,365,729	-1,596,454	-3.63	-0.30
Intact	18,260,940	16,192,952	-2,067,988	-11.32	-0.94
Degraded	25,701,243	26,172,777	+471,534	+1.83	+0.15
Non-Forest	21,134,373	22,122,214	+987,841	+4.67	+0.39
Plantation	917,361	1,453,199	+535,838	+58.41	+4.87
Water	785,771	858,475	+72,704	+9.25	+0.77
Snow/Ice/Clouds	108,684	108,684	0	0	0

*for category definition see Table 1

**sum of Intact and Degraded

**Fig 2:**
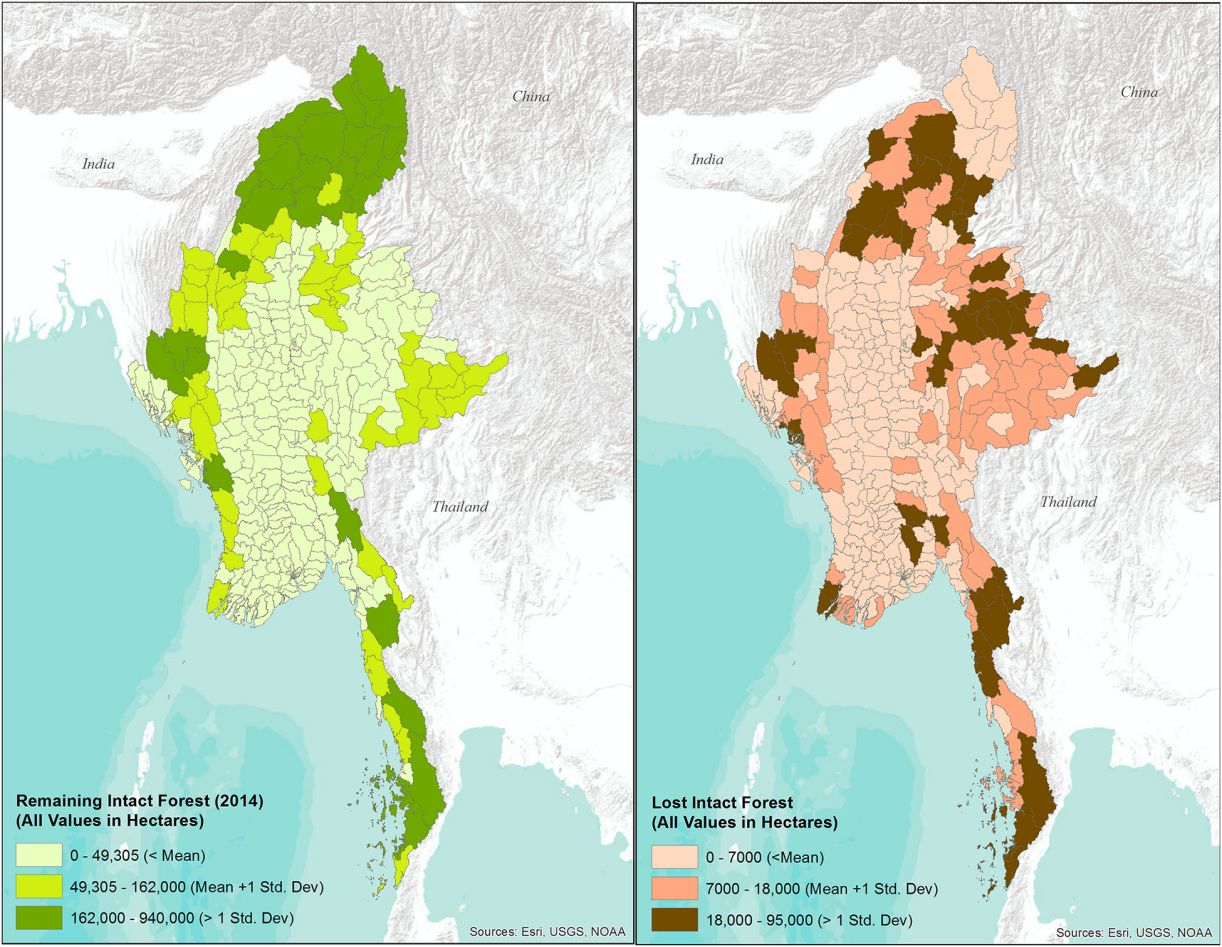
Remaining and lost intact forest (ha) for Myanmar townships between 2002 and 2014.

Most intact forests are concentrated in Myanmar’s hill and mountainous regions, including Kachin, Sagaing, Tanintharyi, Shan, and Chin (13,741,812 ha; 85% of all intact forest; Table 3, Fig 1). Intact forest losses are concentrated in these most forested regions (Fig 2).

**Table 3:** Intact forest cover change by State/Region in Myanmar between 2002 and 2014.

State/Region	Intact Forest [ha]		Intact Forest Loss
	2002	2014	[ha]
Ayeyarwady	267,017	206,935	60,082
Bago	337,230	233,062	104,168
Chin	1,451,943	1,338,413	113,530
Kachin	5,341,463	5,132,416	209,047
Kayah	114,235	96,161	18,074
Kayin	813,934	670,901	143,033
Magway	283,285	219,734	63,551
Mon	156,115	74,731	81,384
Rakhine	856,472	747,448	109,024
Sagaing	3,471,532	3,191,671	279,861
Shan	2,410,844	1,778,238	632,606
Tanintharyi	2,487,026	2,301,074	185,952
Yangon	22,816	12,754	10,062
Mandalay	181,542	141,161	40,381
Naypyitaw	65,486	48,253	17,233

Shan and Sagaing experienced the highest overall losses in intact forest (Table 3). These areas were previously fragmented and probably are exposed to very high pressure from surrounding agricultural land use. However, declines were also very high in more remote and inaccessible areas such as Kachin and Tanintharyi, as well as in some of the other hill regions, including Chin, Bago, Kayin, and Rakhine.

### Forest change hotspots

Using a township analysis, we identified 9 local hotspots for intact forest loss (>18,000 ha) (Table 4, Fig 2). Homalin (87,116 ha), Bokepyin (55,578 ha) (Fig 1D) and Hpakant (39,089 ha) (Fig 1B) lost the highest amount of intact forest between 2002 and 2014. Other notable hotspots included large-scale forest clearing for plantation around Myitkyina along the Ayeyarwady River (Fig 1C) and around Tanai. High losses also occurred in some of the less forested regions (Fig 2), such as the mangrove forests of the Ayeyarwady Delta region in the southwest, and already fragmented forests bordering Laos and China (Fig 2) on the Shan Plateau in the east.

**Table 4:** Deforestation hotspots between 2002 and 2014.

State	Township	Intact forest cover 2002	Intact forest cover 2014	Intact forest cover change in ha	Intact forest cover change in % per year
Chin	Matupi	308,132	285,755	-22,377	0.61
Kachin	Hpakant	398,764	359,675	-39,089	0.82
Kachin	Myitkyina	257,737	233,270	-24,467	0.79
Kachin	Tanai	986,402	948,807	-37,595	0.32
Kachin	Waingmaw	258,896	234,279	-24,617	0.79
Sagaing	Homalin	594,158	507,042	-87,116	1.22
Sagaing	Lahe	283,799	260,152	-23,647	0.69
Tanintharyi	Bokpyin	490,181	434,603	-55,578	0.94
Tanintharyi	Tanintharyi	841,929	821,307	-20,622	0.20

### Plantation development and expansion

Plantation development and expansion reduced intact forests by over 0.54 million ha over the course of our study (Tables 2 & 5) representing >26% of all the intact forest loss. Shan state had the highest total area in plantation use in 2014 (38%) and also experienced the largest expansions (Table 5). An additional 48% of Myanmar’s plantations are concentrated across Kayin State, Tanintharyi Region, Kachin State and Mon State. Plantation expansion in each of these areas was >70,000 ha between 2002 and 2014. All other States/Regions had significantly less area of existing or expanding plantations.

**Table 5:** Plantation area and expansion by State/Region in Myanmar between 2002 and 2014.

State/Region	Plantations 2002 (ha)	Plantations 2014 (ha)	Increase (ha)
Shan	395,919	558,453	162,534
Kayin	66,365	157,650	91,285
Tanintharyi	97,234	172,674	75,440
Kachin	109,291	183,622	74,331
Mon	107,473	181,165	73,692
Sagaing	24,126	42,370	18,244
Bago	49,335	61,723	12,388
Magway	1,608	12,472	10,864
Ayeyarwady	23,073	27,754	4,681
Mandalay	29,151	33,595	4,444
Naypyitaw	986	4,439	3,453
Yangon	4,629	7,674	3,045
Rakhine	4,621	5,775	1,154
Chin	450	733	283
Kayah	3,100	3,100	0

### Remaining intact forest landscapes

**Fig 3:**
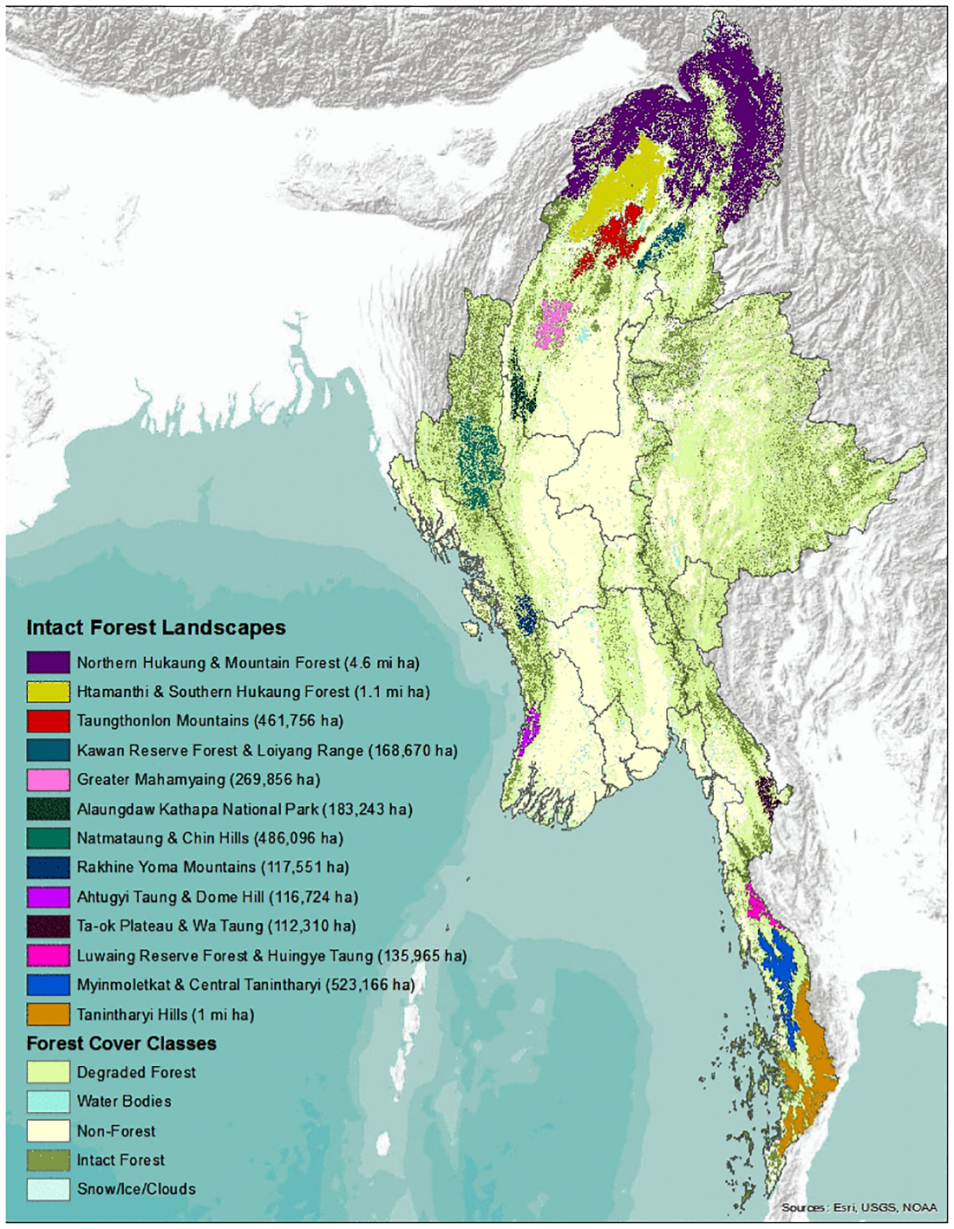
Remaining large and intact forest landscapes in Myanmar 2014.

In 2014, 13 large (>100,000 ha) unfragmented landscapes of intact forest existed in Myanmar (Fig 3). In the north and south these landscapes are clustered forming the Northern and Southern Forest Complexes. There are four large forest landscapes that can be considered part of the Northern Forest Complex and they include the Northern Hukaung and Mountain Forest, the Htamanhti and Southern Hukaung Forest, the Taungthonlon Mountains, and the Lawan Reserve Forest and Loiyang Range (Fig 3). Although these individual landscapes differ in elevation, topography and landform, together they represent one of the largest areas of remaining intact forest in Asia, jointly covering over 6.3 million ha. The Southern Forest Complex links three large and intact forest landscapes, including the Tanintharyi Hills, the Myinmolekat & Central Tanintharyi, and the Luwaing Reserve Forest and Hiungye Taung, jointly covering 1.7 million ha of intact forest. More isolated forest landscapes generally represent areas already included in Myanmar’s protected areas network, such as Alaungdaw Kathapa National Park and adjacent areas along the Natmataung and Chin Hills (Fig 3).

### Accuracy assessment

Classification accuracy decreased with patch size, with a nearly 80% accuracy for very large patches but only a 50% accuracy for very small patches (Table 6). Previous research has shown similar results, indicating that high spatial heterogeneity and small patch size will result in much lower classification accuracies [52]. Using only large patches (100–5,000 ha; 209 patches), the overall accuracy of the forest cover change map was 74% (Table 7). Intact forest was sometimes falsely assigned to degraded forest patches, leading to overestimation of intact forest and underestimation of degraded forest. However, user’s accuracies for intact, degraded and non-forest were high for large patches. Our accuracy values are comparable or better to what has been reported by GlobCover [53]. In comparison to GlobCover our data provides a finer-scale, more recent assessment of remaining forest cover in Myanmar and how much of this forest has been converted.

**Table 6:** Percent agreement between land cover visually assigned to patches and category assigned in the forest cover change map for Myanmar.

Patch Area (ha)	Patches Assessed	Agreement
1–99	351	50.0%
100–499	135	70.4%
500–5,000	74	79.7%

**Table 7:** Error matrix and category accuracies based on a patch-based (100–5,000 ha) accuracy assessment of the forest cover change map for Myanmar. Using Google Earth patches we visually assigned a majority forest cover for patches and compared it to the map categories from the forest cover change map. We only used Google Earth imagery for 2013–14.

Category	Google Earth reference land cover				User’s Accuracy
	Intact	Degraded	Non-Forest	Plantation	
Intact	24	10	0	0	**70.6%**
Degraded	4	35	3	1	**81.4%**
Non-Forest	0	11	63	3	**81.8%**
Plantation	2	9	12	32	**58.2%**
**Producer’s Accuracy**	**80%**	**54%**	**81%**	**89%**	

Although, we found much lower user’s accuracy for plantations, indicating over-prediction, producer’s accuracies were high for this category. This means that if analysts detected a plantation in the Google Earth images, it was generally correctly classified in the forest cover map.

## Discussion

### Intact forest loss

Intact forest cover loss in Myanmar has accelerated over the last decade. Leimgruber et al. [10] found a 0.30% annual rate of forest loss between 1990 and 2000. In our study, overall forests loss rates rose to 0.55% annually. Deforestation and degradation were most pronounced in Myanmar’s valuable intact forests, with annual loss rates of 0.95% and an 11% decrease in total intact forest between 2002 and 2014. Additionally, degraded forests continue to decrease at 0.29% annually, a rate above the global average for forests of 0.13% [54].

Despite the declines, Myanmar continues to be one of the most forested countries in the region. This is encouraging as it indicates that with improved management, the country may be able to rely on renewable forest resources for local subsistence as well as for sustainable commercial use. Currently, forest management practices do not encourage forest restoration or the reclamation of degraded forestlands into plantations and sustainable agricultural use [55]. Remaining intact forests are being exploited for maximum short-term gain at the cost of long-term sustainability. For example, large portions of mangrove forest were converted by aquaculture industries between 1990 and 2010 [56]. Additionally, farmers seem to be adapting to political and economic reforms by **shifting from agriculture to commercially more viable and beneficial cash crops like rubber** [55].

Spatial patterns of intact forest loss in Myanmar are complex. Not surprisingly, the most forested States/Regions experience some of the sharpest reductions, often several 100,000 ha over the course of our study (Table 3, Figs 1 & 2). Declines generally increase if forests are fragmented and already surrounded by agriculture. This can be seen in Shan, where almost all of the remaining intact forest is severely fragmented and overall loss was highest of any State/Region. Similar patterns can be observed in the historically forested regions of Chin, Bago, Kayin, and Rakhine.

The remote areas of Kachin, Sagaing, and Tanintharyi have very large tracts of intact forest remaining. All three State/Regions have experienced substantial forest losses, usually along major river systems, newly constructed highways, or near existing development areas, such as commercial plantations (Fig 1B, 1C & 1D). Similar patterns have previously been observed in the Amazon and Congo Basins [57] where nationally and internationally funded infrastructure and development projects paved the way for increased access and subsequent dramatic forest losses. In Myanmar, these patterns can be especially seen along the Uru and Chindwin Rivers in Sagaing region, as well as along the new roads and pipelines crossing from Thailand into southern Myanmar.

### Major land conversions of intact forest

Some of the primary drivers of deforestation in Myanmar include expansion of large commercial plantations (e.g. oil palm, rubber, sugar cane), agricultural conversion, reliance on fuelwood collection in rural communities, mining, and incursion into forestlands for small-scale permanent agriculture (including small plantation gardens for oil palm, rubber, and sugar cane), and shifting agriculture [58]. Based on our data and analysis, it is not possible to evaluate the role of small-scale agricultural conversion and shifting agriculture for forest management and conservation in Myanmar. Large portions of these areas may also have been degraded by overexploitation for commercial logging and illegal logging. Given the complexity of this issue from social, socioeconomic, forest rights and forest management perspectives, future research and more detailed mapping is critical. While it is difficult to pinpoint the relative contributions of all conversion mechanisms, we can quantify changes caused by large plantation development and expansion, as well as open-pit mining based on changes in spectral reflectance, spatial patterns and shapes, location, and context (Table 2).

Most large-scale plantations in Myanmar grow rubber, sugar cane or palm oil. While total plantation area is still relatively limited, expansion is concentrated in a few States/Regions with high impacts on forest ecosystems and biodiversity. A good example are oil palm plantations in southern Tanintharyi, the only State/Region that has the climate and conditions for their large-scale development and management. Bokpyin and Tanintharyi townships jointly lost more than 75,000 ha of intact forest to oil palm plantation establishment between 2002 and 2014. We also found increases in oil palm plantations in Mawdaung, and along Lenya River (Fig 1).

While oil palm plantations only occur in Tanintharyi, other types of plantations such as rubber, betel nut, banana, and sugar cane are expanding throughout the country. Rubber is a rapidly expanding cash crop throughout the tropical and subtropical areas, and it can grow throughout much of Myanmar. Rubber plantation areas in Northeastern Myanmar, especially Kachin and Shan states cover an area of approximately 70,000 ha [59]. Myitkyina township in Kachin experienced large-scale change to plantations (rubber, banana and cassava), resulting in the loss of about 25,000 ha of intact forest between 2002 and 2014 (Fig 1C).

Illegal, open-pit mining in northern Myanmar, often along streams and rivers, is another major cause of intact forest declines. For example, most of the deforestation along the Uru and Chindwin River in Homalin township was caused by illegal surface mining (Fig 1B). Mining has negative impacts for local communities and biodiversity [52]. Mining alters river flows and can introduce contaminants such as cyanide and mercury. These pollutants accumulate in freshwater fish and their impact cascades through food chains, with the potential to severely affect human health [60].

Shifting cultivation is common in many regions of South and Southeast Asia, as well as in Myanmar. Shifting cultivation produces highly heterogeneous forest landscapes that are characterized by fine-scale variation in forest successions. These characteristics make is near impossible to accurately map shifting cultivation when using mid-resolution imagery such as provided by the Landsat program ([61,62] but see: [63]). Based on our own personal observations in the field (PL & MS), we believe that deforestation of intact forest caused by shifting cultivation generally was classified as degraded forest with no designation of cause. Mapping of these and other small scale deforestation patterns in future will require remotely sensed data with finer spatial and temporal resolution or multi-step thresholding [63].

### Future of Myanmar’s forests and their biodiversity

Much of Myanmar’s remaining intact forests are unprotected and are not included in the country’s reserve forests [64,65]. This may make these forests especially vulnerable to unchecked exploitation, degradation, and broad-scale conversion to permanent agriculture and other developments. Large intact forest complexes in Northern Myanmar such as Northern Hukaung & Mountain Forest and Htamanthi & Southern Hukaung forest are especially vulnerable to forest changes due to unsustainable logging [56], mining expansions, and plantations. These areas have many important and endemic species and surveys in past decades have resulted in the discovery of many new species [15]. Among them are mammals, including leaf deer (*Muntiacus putaoensis*) and Kachin woolly bat (*Kerivoula kachinensis*), several new species of frog (such as *Bufo crocus*) and a new snake (*Naja mandalayenis*) [15].

In Southern Myanmar, unchecked and continued growth of oil palm, may pose a serious threat to the remaining large intact forests, specifically for the Myinmolektat and Tanintharyi Hills landscapes (Fig 3). Both of these areas provide critical habitat for several endangered species [15], including tigers, elephants, Gurney’s pitta, and tapir. In general, oil palms are a poor replacement for intact forest because they support fewer species and cause habitat fragmentation [34]. This has been shown by research on the impacts of oil palm development in Thailand which predicted a severe decline in biodiversity, especially for globally threatened bird species [66,67]. Tanintharyi’s lowland forests currently support the largest remaining populations of Gurney’s pitta in the world. Habitat loss from oil palm may seriously compromise the long-term conservation of this critically endangered bird species in Myanmar [66,67]. Similarly, new oil palm plantations are fragmenting Asian elephant habitats, increasing human-elephant in Tanintharyi. As elephant habitats shrink, conflict will intensify with the potential for serious economic impacts for people, as well as increased human and elephant deaths.

We delineated 13 unfragmented forest landscapes which support much of what is left of Myanmar’s biodiversity, and are strongholds for many highly endangered species such as Gurney’s pitta and tiger (Fig 3). Large portions of these landscapes should be included in Myanmar’s National Biodiversity Strategy [15]. While it may not be possible to include all of these areas into the country’s growing protected area system, future management of these forests should be carefully planned and monitored. Conversion of large stretches to commercial cash crops should be minimized in favor of sustainable harvesting of high-value timber with long rotation cycles.

Myanmar is at a crossroads where overall forest cover remains high, but intact forest is quickly being lost or degraded. Protection of remaining intact forests, and restoration of degraded forest are critical to ensuring the long-term future of the country’s forests. Both sustainable forest use and conservation of intact forest ecosystems are essential components for ensuring the health and economic development of Myanmar’s people. This requires substantial reforms, including the development of comprehensive land reforms, land use planning, the creation of sustainable forest policies and management, and improving governance.

Many of these processes have already been initiated but it is unclear how far-reaching and successful these reforms will be. Access to up-to-date and accurate spatial data on forest condition is critical. Unfortunately, data on Myanmar’s natural resources seems to be either very limited or not accessible to civil society and the public. Our project demonstrates that grassroots efforts that utilize open source data and tools for forest mapping can produce new and accurate information to assess the current status of Myanmar’s forests. By putting our data into public domain, independent review of our results is possible and we hope will result in additional and expanded assessments of Myanmar’s forests and the processes that affect their future.

### Comparison with existing data sets

To put our mapping approach into context, we compared our results with the numbers reported by the Food and Agriculture Organization’s (FAO) Global Forest Resource Assessment (FRA) for Myanmar [67]. The FRA defines forest as all areas with canopy cover >10%, which corresponds to our combined intact and degraded forests. However, our intact forest definition is stricter than FAO’s closed forest, with a threshold of 80% compared to the FRA threshold of 40% canopy cover [68]. During our pre-mapping workshop, forest experts agreed that most forest types in Myanmar, including evergreen, mixed deciduous-evergreen, mangrove, and bamboo have very well developed and dense canopies, with canopy cover well above 80%. Forests below these cover values are degraded, either by recent clear-cutting, overuse, fuelwood extraction or conversion to plantations. Consequently, much of the forests categorized by FAO as closed and open canopy forest, fall into our degraded category (see S2 Table for a more detailed comparison). The only exception from this rule are dry deciduous, dry dipterocarp, and thorn forests which occur in small patches along the edges of the central dry zone. These forest types are naturally relatively open, but usually not below 60% canopy cover [41,42,44]. FRA forest (sum of closed and open canopy forests and other wooded lands) and forest areas in our map (sum of intact and degraded forest) show close agreement (S2 Table). The differences between our results lie in the differentiation of open forest vs. degraded forest. Differences in our study and the FAO’s FRA 2015 report stem from our decision to place a major focus on delineating intact forests, i.e. closed canopy forests with a canopy cover >80%. This definition is much more restrictive than FAO’s closed canopy forest definition [68] (S2 Table). This is justified because we wanted to be able to quantify and map the location of Myanmar’s best remaining forests. These data now provide a baseline against which the success of future land use planning, protection efforts, and sustainable forestry policies can be measured.

Another significant difference between FAO’s report and our study lies in the treatment of open canopy forests and other woodlands. Most of Myanmar’s forests, with the exception of a small area of dry dipterocarp forests, are closed canopy forest types. Although these savanna type forests may have been a dominant forest type in Myanmar’s central dry zone historically, they have been almost completely replaced by agriculture and only small areas are left. All other open canopy forests, must be considered degraded forests. Labeling these forests as open canopy creates the false impression that they can currently be utilized in sustainable forestry. These degraded forests are very vulnerable to additional degradation, especially from encroachment by people and overuse for fuelwood. New forestry policies and strategies are needed to restore degraded forests.

**Fig 4:**
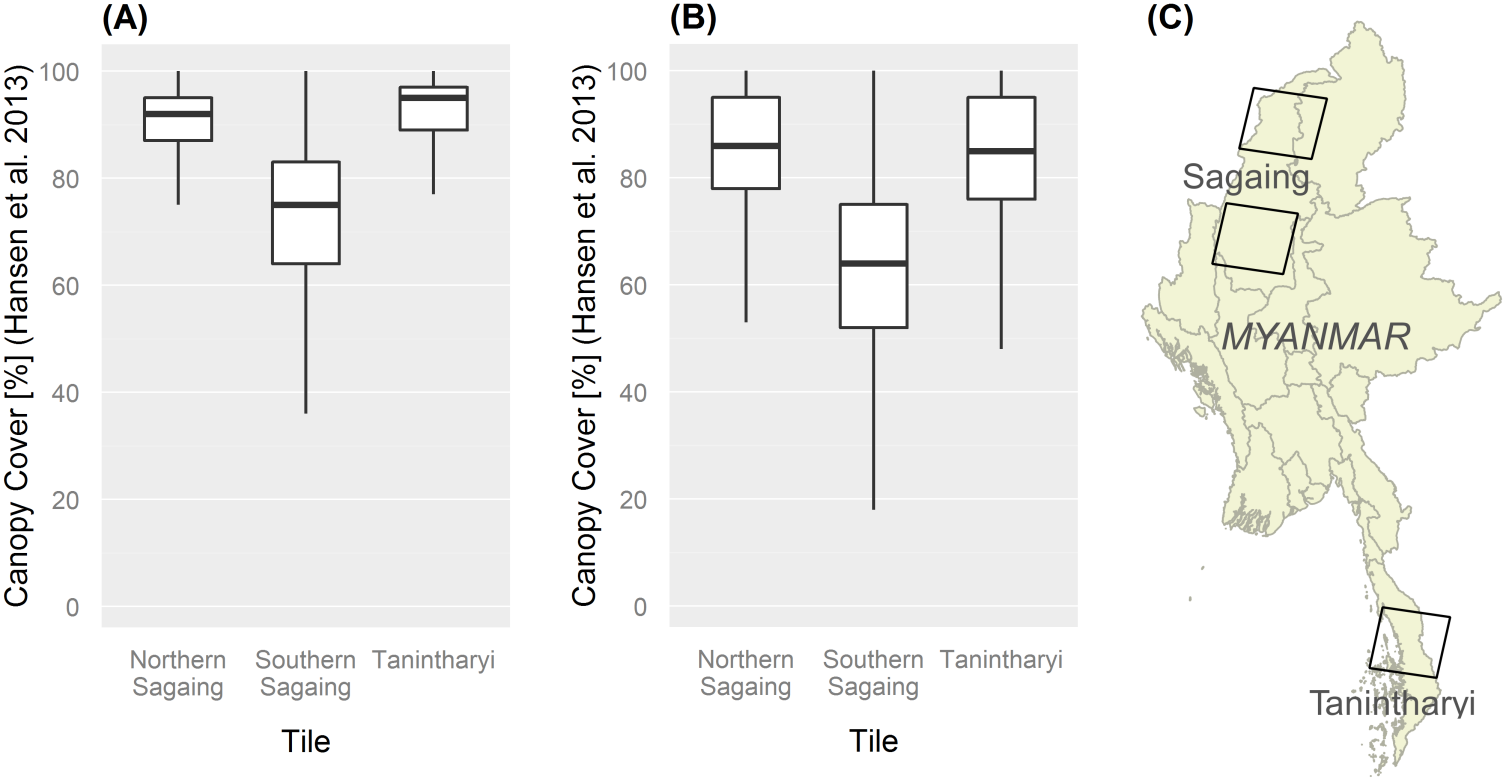
Boxplot comparing mean canopy cover as measured by Hansen et al. (2013) for intact and degraded forests in different forest regions of Myanmar between 2002 and 2014. Northern Sagaing = mostly mixed deciduous and evergreen forest; Southern Sagaing = mixed deciduous and dry dipterocarp forest; Tanintharyi = tropical evergreen forest. (A) Canopy cover values for intact forest; (B) Canopy Cover values for degraded forest; (C) Location of Landsat tiles used for comparison.

We also used percent canopy data provided by Hansen et al. [26] to compare canopy between three distinct forest regions of Myanmar, including upland broadleaf and evergreen forests in Kachin, dry deciduous and dry dipterocarp forest in Saigang, and lowland Sundaic evergreen forest in Tanintharyi. The purpose of this comparison was to ensure that a) we consistently mapped intact forests across Myanmar from North to South, and that b) our relaxation of the cut-off (60% vs. 80%) for areas with dry deciduous and dry dipertocarp forests produced reasonable results. We selected three Landsat scenes and extracted values from Hansen et al.’s percentage tree cover dataset from 2000 [26] for areas we classified as a) intact forest and b) degraded forest. The results confirmed that our technique was well suited to delineate intact forests with >80% canopy cover (Fig 4). Average tree cover values derived from Hansen et al. [26] are 91% for intact forest in Northern Sagaing and Tanintharyi (median 95% and 92%; Fig 4A), compared to means of 78% and 79% for degraded forest (median 85% and 85%) (Fig 4B). In Southern Sagaing, where dry dipterocarp forests are common, intact forest showed 72% tree cover on average (median 75%), while the mean was 57% in degraded forest (median 63%).

#### Challenges in using remote sensing

Analyzing satellite imagery is an effective and established tool to quantify land cover and land use, especially changes in forest and canopy cover [19–23,69], but also poses challenges, especially in the tropics. Cloud cover can be an obstacle in the humid tropics, which we overcame by using Landsat imagery from a time of the year with low cloud cover and filling in areas of cloud cover using imagery from different time period. A second challenge is quantifying ongoing forest degradation. While we focused our analysis on intact forest we also show that large areas of forest in Myanmar are already degraded. Degradation can impact much larger areas than active deforestation and contributes to biodiversity loss and carbon emissions [70,71]. Accurately mapping degradation is challenging due the variety of degradation causes and human impacts, availability of data as Landsat imagery and technical difficulties to distinguish small differences in canopy cover [72].

#### Map accuracy

Assessing classification accuracy for large area mapping and change mapping is challenging because frequently there is little data available for an independent evaluation of the mapping results. Comparisons of our outputs with other existing data sets [26,68] show high agreement when taking into account differences in the definition of the forest categories. Neither of these data provide accuracy information, yet they are used extensively to assess forest condition for Myanmar and the region.

We decided to include an accuracy assessment that was a) matched to the country-wide scale of our study, and b) focused on our ability to accurately map forest patches (= polygons). We focused this assessment on our 2013–2014 map, because of the lack of high resolution imagery for the 2002–2003 time period. With evaluation data from only one time step, we were also not able to determine accuracies for change categories. However, we were able to show that our 2013–2014 forest categories are 74% accurate when looking at the largest patches, which we believe is acceptable. This is especially true for our intact forest category which had high user’s and producer’s accuracies (Table 6). Predicting large patches of remaining intact forest with high accuracy is especially important because these areas need to be prioritized for improved forest management and conservation.

Low accuracy of small patches (1–99 ha) has previously been reported [52]. These patches tend to be in areas with high spatial heterogeneity. Accuracy for smaller patches could probably improve locally but is difficult to improve when the goal is to map an entire country.

Class accuracy was slightly reduced because we over-estimated intact forest as well as plantation area. Some overestimation of intact forest may have been caused by topographic shading in mountainous areas. For intact forests this is acceptable, because one of the main goals in our study was to identify and map remaining intact forests for future conservation.

Plantations are difficult to map using mid-resolution satellite images, but also can be difficult to detect by visual inspection of Google Earth imagery alone. It’s possible that that the lower accuracies may also be affected by lower accuracy of identifying some plantation types (e.g. rubber, cashew) in Google Earth. Other classification errors may be due to mismatches between the Landsat and Google Earth imagery dates, smoothing of the raster maps, or topographic shadows.

## Supporting information

S1 Table Landsat scenes. Landsat scenes and tiles used in the analysis.
(DOCX)

S2 Table Comparison to the Forest Resource Assessment 2015 –Country report, Myanmar. (DOCX)

S1 Fig Example feature space plots. Analysist used feature space plots (FSP) to cross-check confusion between different forest cover categories. FSPs were plotted comparing the values for near-infrared bands for different forest cover categories (Layer 5 = near-infrared in 2002; Layer 11 = near-infrared in 2014, 1 = Intact forest, 2 = changed forest, 3 = water, 4 = other).
(DOCX)

S1 File R scripts for random forest. (DOCX)
