# Supplementary material for: Losing a jewel—Rapid declines in Myanmar’s intact forests from 2002-2014
Source: PLoS One. 2017 May 17;12(5):e0176364. doi: 10.1371/journal.pone.0176364 (PMC5435175; doi:10.1371/journal.pone.0176364)
Supplement: S1 Table — Landsat scenes and tiles used in the analysis. (DOCX) [file pone.0176364.s001.docx]

**S1. Landsat Scenes.** Landsat scenes and tiles used in the analysis.

|  | Primary Classification | | Cloud Classification | |
| --- | --- | --- | --- | --- |
| Tile | Early Time  (Landsat 5,7) | Late Time  (Landsat 8) | Early Time  (Landsat 5,7) | Late Time  (Landsat 8) |
| 129_52 | LE71290522001325 | LC81290522014033 | LE71290522001325 | LC81290522014033 |
| 130_45 | LE71300452003050 | LC81300452014040 | LE71300452003050 | LC81300452014040 |
| 130_46 | LE71300462003050 | LC81300462014040 | LE71300462003066 | LC81300462014040 |
| 130_50 | LE71300502003018 | LC81300502014024 | LE71300502003018 | LC81300502014024 |
| 130_51 | LE71300512003362 | LC81300512013357 | LE71300512003362 | LC81300512013357 |
| 130_52 | LE71300522000362 | LC81300522013357 | LE71300522000362 | LC81300522013357 |
| 130_53 | LE71300532003018 | LC81300532014024 | LE71300532003018 | LC81300532014024 |
| 131_44 | LT51310442001043 | LC81310442014031 | LE71310442003057 | LC81310442014031 |
| 131_45 | LE71310452003057 | LC81310452014047 | LE71310452002086 | LC81310452015050 |
| 131_46 | LE71310462003057 | LC81310462014047 | LE71310462003057 | LC81310462014047 |
| 131_48 | LE71310482002310 | LC81310482013316 | LE71310482003041 | LT51310482011039 |
| 131_49 | LE71310492002310 | LC81310492013348 | LE71310492003041 | LC81310492014015 |
| 131_50 | LE71310502002310 | LC81310502013316 | LE71310502002310 | LC81310502013316 |
| 132_41 | LT51320412001354 | LC81320412013339 | LT51320412001354 | LC81320412013339 |
| 132_42 | LT51320422001354 | LC81320422013339 | LT51320422001354 | LC81320422013339 |
| 132_43 | LE71320432002365 | LC81320432013339 | LE71320432003016 | LC81320432015073 |
| 132_44 | LE71320442003048 | LC81320442014054 | LE71320442003048 | LC81320442014054 |
| 132_45 | LE71320452003064 | LC81320452014054 | LE71320452002061 | LC81320452015073 |
| 132_46 | LE71320462003032 | LC81320462014038 | LE71320462002061 | LC81320462014038 |
| 132_47 | LE71320472000312 | LC81320472013355 | LE71320472003032 | LC81320472014086 |
| 132_48 | LE71320482000312 | LC81320482013307 | LE71320482001362 | LC81320482013355 |
| 132_49 | LE71320492003032 | LC81320492013323 | LE71320492002349 | LC81320492014054 |
| 133_40 | LE71330402001337 | LC81330402013330 | LE71330402001337 | LC81330402013330 |
| 133_41 | LT51330412004322 | LC81330412013314 |  |  |
| 133_42 | LT51330422004306 | LC81330422013314 | LE7133422003055 | LC8133422013314 |
| 133_43 | LE71330432003055 | LC81330432014045 |  |  |
| 133_44 | LE71330442003055 | LC81330442014077 | LE71330442003055 | LC81330442014077 |
| 133_45 | LE71330452003023 | LC81330452014013 | LE71330452003055 | LC81330452014013 |
| 133_46 | LE71330462003023 | LC81330462014013 | LE71330462002052 | LC81330462014061 |
| 133_47 | LE71330472003023 | LC81330472014013 | LE71330472003023 | LC81330472014013 |
| 133_48 | LE71330482003055 | LC81330482014013 | LE71330482003055 | LC81330482014013 |
| 133_49 | LE71330492001001 | LC81330492014013 | LE71330492001065 | LC81330492014029 |
| 134_41 | LE71340412001360 | LC81340412013321 | LE71340412001360 | LC81340412013321 |
| 134_42 | LT51340422004041 | LC81340422014052 | LT51340422004041 | LC81340422014052 |
| 134_43 | LE71340432001296 | LC81340432014036 | LE71340432001296 | LC81340432014036 |
| 134_44 | LE71340442002043 | LC81340442013321 | LE71340442002043 | LC81340442013321 |
| 134_45 | LE71340452001024 | LC81340452014020 | LE71340452001024 | LC81340452013036 |
| 134_46 | LE71340462001328 | LC81340462013321 | LE71340462002059 | LT51340462011044 |
| 134_47 | LE71340472002363 | LC81340472013353 | LE71340472002363 | LC81340472013353 |
| 135_43 | LE71350432002050 | LC81350432013312 | LE71350432004064 | LC81350432014075 |
| 135_44 | LE71350442002322 | LC81350442013312 | LE71350442002059 | LC81350442014075 |
| 135_45 | LE71350452000045 | LC81350452015030 | LE71350452003021 | LC81350452015030 |
| 135_46 | LE71350462000317 | LC81350462013312 | LE71350462002066 | LC813050462014347 |
