# Supplementary material for: Losing a jewel—Rapid declines in Myanmar’s intact forests from 2002-2014
Source: PLoS One. 2017 May 17;12(5):e0176364. doi: 10.1371/journal.pone.0176364 (PMC5435175; doi:10.1371/journal.pone.0176364)
Supplement: S2 Table — (DOCX) [file pone.0176364.s002.docx]

**S2 Table. Comparison to the Forest Resource Assessment 2015 – Country Report, Myanmar.**

| **FRA Category** | **FRA Category Definition** | **Corresponding Categories in Our Study** |
| --- | --- | --- |
| **Closed Forest** | Under forestry or no other land use, spanning more than 0.5 ha; with trees higher than 5 m and a CC*>40%, or trees able to reach these thresholds in situ. | Intact Forest (CC>80% ), Degraded Forest (CC=40-80%), Plantation (CC=40-100%) |
| **Open Forest** | Under forestry or no other land use, spanning more than 0.5 hectares; with trees higher than 5 meters and CC 10-40%, or trees able to reach these thresholds in situ. | Degraded Forest (CC=10-40%), Plantation (CC=10-40%) |
| **Forest** | Land spanning more than 0.5 hectares with trees higher than 5 meters and CC >10% or trees able to reach these thresholds in situ. It does not include land that is predominantly under agricultural or urban land use. The sum of Closed Forest and Open Forest. | Intact Forest, Degraded Forest, Plantation |
| **Other Wooded Land** | Land not classified as "Forest" spanning more than 0.5 hectares with trees higher than 5 meters and CC 5-10% or trees able to reach these thresholds; or with a combined cover of shrubs, bushes, and trees >10%. It does not include land that is predominantly under agricultural or urban land use. | Non-Forest (CC<10%; vegetated, like shrubland), Degraded Forest (i.e. for trees and shrubs with CC> 10%) |
| **Other Land** | All land that is not classified as "Forest" or "Other wooded land". | Non-Forest (bare ground and infrastructure), Snow/Ice |
| **Inland Water Bodies** | Inland water bodies generally include major rivers, lakes and water reservoirs. | Water |

*CC = Canopy Cover
