# Supplementary material for: Losing a jewel—Rapid declines in Myanmar’s intact forests from 2002-2014
Source: PLoS One. 2017 May 17;12(5):e0176364. doi: 10.1371/journal.pone.0176364 (PMC5435175; doi:10.1371/journal.pone.0176364)

**S1 Fig. Example Feature Space Plots.**

Analysist used feature space plots (FSP) to cross-check confusion between different forest cover categories. FSPs were plotted comparing the values for near-infrared bands for different forest cover categories (Layer 5=near-infrared in 2002; Layer 11=near-infrared in 2014, 1=Intact forest, 2=changed forest, 3=water, 4=other).


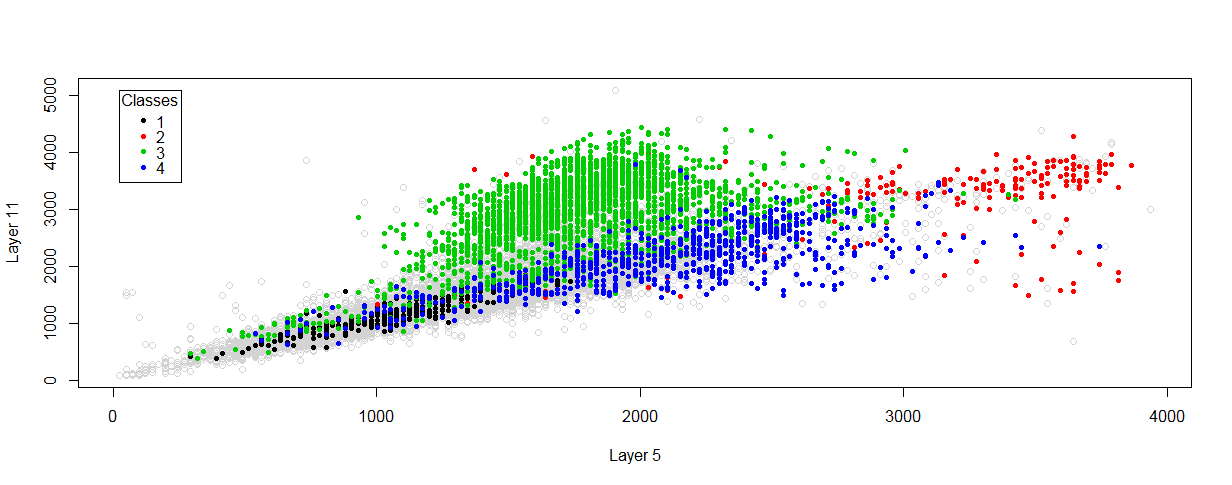

Supplement: S1 Fig — Analysist used feature space plots (FSP) to cross-check confusion between different forest cover categories. FSPs were plotted comparing the values for near-infrared bands for different forest cover categories (Layer 5 = near-infrared in 2002; Layer 11 = near-infrared in 2014, 1 = Intact forest, 2 = changed forest, 3 = water, 4 = other). (DOCX) [file pone.0176364.s003.docx]
