# Supplementary material for: Losing a jewel—Rapid declines in Myanmar’s intact forests from 2002-2014
Source: PLoS One. 2017 May 17;12(5):e0176364. doi: 10.1371/journal.pone.0176364 (PMC5435175; doi:10.1371/journal.pone.0176364)
Supplement: S1 File — (DOCX) [file pone.0176364.s004.docx]

**S1 File. R Scripts for Random Forest.**

The following is the R script we used to classify Landsat tiles of Myanmar using Random Forest. This script was written by Dr. Ned Horning of the American Museum of Natural History, and is also available online with a user guide at: <https://bitbucket.org/rsbiodiv>.

#############################################################################

# The script reads an ESRI Shapefile (defined by the "shapefile" variable) with

# training polygons and then either selects all pixels or randomly selects a

# user-determined number of samples (defined using classNums and classSampNums)

# for each land cover type. A multilayer image that contains spectral, other

# continuous data or categorical data is also input (defined by the inImage

# variable). For each randomly selected sample the data values for that pixel

# are determined and these data are used to run the Random Forest model.

#

# After building the model the multilayer image is read, and up to three output

# images (classImage, probImage, threshImage) can be selected.

# "classImage" classifies all of the pixels.

#

# "probImage" outputs the class probability of the class that got the most votes

# (i.e., the class that was selected for the classImage layer).

#

# "threshImage" is the same as "classImage" except all pixels with a class probability

# of the class that got the most votes below the "probThreshold" parameter are set to 0.

# This is useful to identify pixels with inter-class confusion.

#

# The images are written out using the GeoTIFF format and the file name is created by appending

# "_Class" to the input image file name and it is written to the same directory as the input

# image. A variable importance plot is displayed to provide information

# about the influence of each variable. An error rate estimate and confusion matrix are also

# printed to provide information about classification accuracy.

#

# There is an option to assess the quality of the training data. The metric for this

# is the margin. The margin of a training point is the proportion of votes for the correct

# class minus maximum proportion of votes for the other classes for that segment. Positive margin

# values represent correct classification, and vice versa. The margin data are written to a

# point ESRI Shapefile so they can be overlaid on the image and training polygons to assess which

# points need to be removed and relabeled in the training data and it can help determine which

# classes needs additional training segments. If this output is not needed you can enter two

# double or single-quotes ("" or '') for the variable outPointsFile.

#

# There is also an option to output a feature space plot using two bands of your choice.

# If a feature space plot is not needed then enter "0" for the variables xBand and/or yBand.

# When a feature space plot is drawn it is possible to define a rectange on the plot to highlight

# pixels in the image that are not well represented in the trianing data.

#

# Set the variables below in the "SET VARIABLES HERE" section of the script.

#

# This script was written by Ned Horning [horning@amnh.org]

# Support for writing and maintaining this script comes from The John D. and

# Catherine T. MacArthur Foundation and Google.org.

#

# This script is free software; you can redistribute it and/or modify it under the

# terms of the GNU General Public License as published by the Free Software Foundation

# either version 2 of the Licenase, or ( at your option ) any later version. *

#

#############################################################################

#Load libraries

require(maptools)

require(sp)

require(randomForest)

require(raster)

require (rgdal)

#

cat("Set variables and start processing\n")

#

############################# SET VARIABLES HERE ###################################

# Set working directory

setwd("/home/nedhorning/R_Project/TestData")

# Name and path for the Shapefile (don't need the .shp extension)

shapefile <- '/home/nedhorning/R_Project/TestData/spot_400_train/spot_400_train.shp'

# Class numbers that you want to select fraining sample from

classNums <- c(2,3,4,7)

# For each land cover class the approximate number of training samples to be randomly selected

# If a value is "0" then all pixels in all of the polygons for that classwill be used

classSampNums <- c(500, 500, 200, 600)

# Name of the attribute that holds the integer land cover type identifyer

attName <- 'type_id'

# No-data value for the input image

nd <- 0

# Name and path for the input satellite image

inImageName <-'spot_subset_400.tif'

# Name and location of the output Shapefile point file that will be created. If this output

# is not needed you can enter two double or single-quotes (""?? or '')

# Note that if this file exists the write will fail with the message "Creation of output file failed"

outMarginFile <- 'margin.shp'

# Output classification image (enter TRUE or FALSE)

classImage <- TRUE

# Output probability image layer (enter TRUE or FALSE)

probImage <- TRUE

# Output classification layer and set pixels with probability less than "probThreshold" to 0 (enter TRUE or FALSE)

threshImage <- TRUE

# Enter threshold probability in percent (values must be between 0 and 100) only used if threshImage=TRUE

probThreshold <- 75

# Layer number (band number) for the X and Y axis of the feature space plot.

# If you do not want to calculate a feature plot enter 0 as the layer number

xBand <- 2

yBand <- 3

#######################################################################################

#

# Start processing

startTime <- Sys.time()

cat("Start time", format(startTime),"\n")

# Read the Shapefile

vec <- readShapePoly(shapefile)

# Load the image then flag all no-data values(nd) so they are not processed

satImage <- brick(inImageName)

NAvalue(satImage) <- nd

#for (b in 1:nlayers(satImage)) { NAvalue(satImage@layers[[b]]) <- nd }

# Create vector of unique land cover attribute values

allAtt <- vec@data

tabAtt <-table(allAtt[[attName]])

uniqueAtt <-as.numeric(names(tabAtt))

# Check if lenght of classNums and classSampNums is equal

if (length(classNums) != length(classSampNums)) {

cat("\n***************length of classNums and classSampNums no equal***************** \n")

stop("Check the classNums and classSampNums variable\n", call.=FALSE)

}

# Check if all classNums exist in uniqueAtt

#### CHECK THIS FUNCTION TO SEE IF classNums ARE IN uniqueAtt ################

if (sum(classNums %in% uniqueAtt) != length(uniqueAtt)) {

cat("\n*******not all classes in classNums are defined in the vecotr file******* \n")

stop("Check classNums and vector attribute table\n", call.=FALSE)

}

# Create input data from a Shapefile using all training data

cat("Create training data using all pixels in training polygons\n")

predictors <- data.frame()

response <- numeric()

xyCoords <- data.frame()

cat("Create training data to train model\n")

# If all pixels in a polygon are to be used process this block

for (n in 1:length(classNums)) {

if (classSampNums[n] == 0) {

# Get the metadata for all polygons for this particular class

class_data<- vec[vec[[attName]]==classNums[n],]

# Extract and combine predictor and response variables for each polygon within a class

for (i in 1:dim(class_data)[1]) {

satValues <- extract(satImage, class_data[i,], cellnumbers=TRUE, df=TRUE)

## satValues <- as.data.frame(do.call(rbind,satValues))

attributeVector <- rep.int(classNums[n],nrow(satValues))

xyCoords <- rbind(xyCoords, xyFromCell(satImage, satValues[,2]))

predictors <- rbind(predictors, satValues[,-1:-2])

response <- c(response, attributeVector)

}

} else {

# Create input data from a Shapefile by sampling training data polygons

# Get the metadata for all polygons for a particular class (based on the uniqueAtt variable)

class_data<- vec[vec[[attName]]==classNums[n],]

# Get the area of each polygon for a particular class

areas <- sapply(slot(class_data, "polygons"), slot, "area")

# Calculate the number of samples for each polygon based on the area in proportion to total area for a class

nsamps <- ceiling(classSampNums[n]*(areas/sum(areas)))

# Use random sampling to select training points (proportial based on area) from each polygon for a given class

for (i in 1:dim(class_data)[1]) {

xy_class <- spsample(class_data[i,], type="random", n=nsamps[i])

# Add coordinates to create a list of random points for all polygons

if (i == 1) cpts <- xy_class

else cpts <- rbind(cpts, xy_class)

}

# The number of points might not match numsamps exactly.

xy_ForClass <- cpts

xyCoords <- rbind(xyCoords, xy_ForClass@coords)

# Get class number for each sample point for responce variable

response <- c(response, over(xy_ForClass, vec)[[attName]])

# Get pixel DNs from the image for each sample point

predictors <- rbind(predictors, extract(satImage, xy_ForClass))

}

}

trainvals <- cbind(response, predictors)

# Test if feature space plot is needed

if (xBand != 0 & yBand != 0) {

#Plot feature space and samples

continue <- "c"

while (continue == "c") {

plotImage <- stack(satImage[[xBand]], satImage[[yBand]])

# Get pixel values from the image under each sample point and create a table with

# observed and predicted values

cat("Getting pixel values to create feature space plot\n\n")

featurePlotPoints <- sampleRegular(plotImage,100000 )

# Remove NA values from trainvals table created above

featurePlotPoints <- na.omit(featurePlotPoints)

minBand1 <- min(featurePlotPoints[,1])

maxBand1 <- max(featurePlotPoints[,1])

minBand2 <- min(featurePlotPoints[,2])

maxBand2 <- max(featurePlotPoints[,2])

rangeBand1 <- maxBand1 - minBand1 + 1

rangeBand2 <- maxBand2 - minBand2 + 1

xAxisLabel <- paste("Layer", xBand, sep=" ")

yAxisLabel <- paste("Layer", yBand, sep=" ")

plot(featurePlotPoints[,1], featurePlotPoints[,2], col="lightgrey", xlab=xAxisLabel, ylab=yAxisLabel)

uniqueValues <- unique(trainvals[,1])

for (v in 1:length(uniqueValues)) {

points(trainvals[which(trainvals[,1]==uniqueValues[v]), xBand+1], trainvals[which(trainvals[,1]==uniqueValues[v]), yBand+1], col=v, pch=20)

}

legend(minBand1, maxBand2, col=1:v, pch=20, title="Classes", legend=as.character(uniqueValues))

continue <- readline(prompt="Type n to stop, c to change feature space bands, s to define a rectangle to locate gaps in feature space, or any other key to continue with randome forests model creation and prediciton: \n\n")

if (substr(continue, 1,1) == "n") {

stop("Processing stopped at users request \n\n", call.=FALSE)

}

if (substr(continue, 1,1) == "s") {

cat("Click two points to define the area on the feature space plot that you want to highlight\n")

coords <- locator(n=2)

coords <- unlist(coords)

xvals <- coords[1:2]

yvals <- coords[3:4]

# Print out the corner coordinates for the rectangle

cat("min X =", min(xvals), "\n")

cat("max X =", max(xvals), "\n")

cat("min y =", min(yvals), "\n")

cat("max y =", max(yvals), "\n")

# Draw the rectangle on the feature space plot

rectangle <- matrix(nrow=5, ncol=2)

rectangle[1,] <- c(min(xvals), max(yvals))

rectangle[2,] <- c(max(xvals), max(yvals))

rectangle[3,] <- c(max(xvals), min(yvals))

rectangle[4,] <- c(min(xvals), min(yvals))

rectangle[5,] <- c(min(xvals), max(yvals))

lines(rectangle[,1], rectangle[,2])

# Get the bands used to calculate the feature space plot

b1 <- raster(plotImage, layer=1)

b2 <- raster(plotImage, layer=2)

# Threshold satImage so all values selected in the rectangle on the feature space plot are set to 255

satImage[(b1 > min(xvals)) & (b1 < max(xvals)) & (b2 > min(yvals)) & (b2 < max(yvals))] <- 255

# Plot the thresholded image with selected pixels displayed as white pixels

plotRGB(satImage, r=1,g=2,b=3, , stretch='hist')

cat("White pixels in the plotted image were selected in the rectangle drawn on the feature space plot")

stop("Add new training data and re-run the script \n\n", call.=FALSE)

}

if (substr(continue, 1,1) == "c") {

xBand <- as.numeric(readline(prompt="Enter the band number for the x axis: \n"))

yBand <- as.numeric(readline(prompt="Enter the band number for the y axis: \n"))

}

}

}

# Remove NA values

trainvals <- na.omit(trainvals)

# Check to make sure Shapefile and input image are in the same projection

if (nrow(trainvals) == 0) {

cat("\n*************************No training data found**************************** \n")

stop("It is possible the projection of the Shapefile with training data and input image are different\nCheck projections and run again", call.=FALSE)

}

# Run Random Forest

cat("Calculating random forest object\n")

randfor <- randomForest(as.factor(response) ~., data=trainvals, importance=TRUE, na.action=na.omit)

# Start predictions

cat("Starting predictions\n")

# Calculate the image block size for processing

bs <- blockSize(satImage)

extensionName <- unlist(strsplit(inImageName, "\\."))[length(unlist(strsplit(inImageName, "\\.")))]

outFileBaseName <- unlist(strsplit(inImageName, paste("\\.", extensionName, sep="")))[1]

# Create the output rasters

if (classImage) {

outClassImage <- raster(satImage)

outClassImage <- writeStart(outClassImage, filename=paste(outFileBaseName, "_Class.tif", sep=""), navalue=0, progress='text', format='GTiff', datatype='INT1U', overwrite=TRUE)

}

if (probImage) {

outProbImage <- raster(satImage)

outProbImage <- writeStart(outProbImage, filename=paste(outFileBaseName, "_Prob.tif", sep=""), navalue=0, progress='text', format='GTiff', datatype='INT1U', overwrite=TRUE)

}

if (threshImage) {

outThreshImage <- raster(satImage)

outThreshImage <- writeStart(outThreshImage, filename=paste(outFileBaseName, "_Thresh.tif", sep=""), navalue=0, progress='text', format='GTiff', datatype='INT1U', overwrite=TRUE)

}

# Loop though each of the image blocks to calculate the output layers selected in the variables section

for (i in 1:bs$n) {

cat("processing block", i, "of", bs$n, "\r")

imageBlock <- getValuesBlock(satImage, row=bs$row[i], nrows=bs$nrows[i])

predValues <- predict(randfor, imageBlock, type='response')

classValues <- as.numeric(levels(predValues))[predValues]

if (classImage) {

#outClassMatrix <- matrix(classValues, nrow=nrow(imageBlock), ncol=1)

outClassImage <- writeValues(outClassImage, classValues, bs$row[i])

}

if (probImage || threshImage) {

predProbs <- as.data.frame(predict(randfor, imageBlock, type='prob'))

maxProb <- round(apply(predProbs, 1, max) * 100)

if (probImage) {

#outProbMatrix <- matrix(maxProb, nrow=nrow(imageBlock), ncol=1)

outProbImage <- writeValues(outProbImage, maxProb, bs$row[i])

}

if (threshImage) {

threshValues <- classValues

threshValues[which(maxProb <= probThreshold)] <- 0

#outThreshMatrix <- matrix(threshValues, nrow=nrow(imageBlock), ncol=1)

outThreshImage <- writeValues(outThreshImage, threshValues, bs$row[i])

}

}

}

# Stop writing and close the file

if (classImage) {

outClassImage <- writeStop(outClassImage)

}

if (probImage) {

outProbImage <- writeStop(outProbImage)

}

if (threshImage) {

outThreshImage <- writeStop(outThreshImage)

}

# Print error rate and confusion matrix for this classification

confMatrix <- randfor$confusion

cat("#################################################################################\n")

cat("OOB error rate estimate\n", 1 - (sum(diag(confMatrix)) / sum(confMatrix[,1:ncol(confMatrix)-1])), "%\n\n", sep="")

cat("Confusion matrix\n")

print(randfor$confusion)

cat("\n")

if (outMarginFile != "") {

# Calculate margin (proportion of votes for correct class minus maximum proportion of votes for other classes)

marginData <- margin(randfor)

trainingAccuracy <- cbind(marginData[order(marginData)], trainvals[order(marginData),1])

# Add column names to attributes table

colnames(trainingAccuracy) <- c("margin", "classNum")

# Order X and Y coordinates

xyCoords <- xyCoords[order(marginData),]

# Create and write point Shapefile with margin information to help improve training data

pointVector <- SpatialPointsDataFrame(xyCoords, as.data.frame(trainingAccuracy), coords.nrs = numeric(0), proj4string = satImage@crs)

writeOGR(pointVector, outMarginFile, "layer", driver="ESRI Shapefile", check_exists=TRUE)

}

# Plotting variable importance plot

varImpPlot(randfor)

# Calculate processing time

timeDiff <- Sys.time() - startTime

cat("\nProcessing time", format(timeDiff), "\n")
